# Prevalence of Prediabetes and Undiagnosed Diabetes in Patients with HFpEF and HFrEF and Associated Clinical Outcomes

**DOI:** 10.1007/s10557-017-6754-x

**Published:** 2017-09-25

**Authors:** Søren L. Kristensen, Pardeep S. Jhund, Matthew M. Y. Lee, Lars Køber, Scott D. Solomon, Christopher B. Granger, Salim Yusuf, Marc A. Pfeffer, Karl Swedberg, John J. V. McMurray, CHARM Investigators and Committees

**Affiliations:** 1 British Heart Foundation Cardiovascular Research Centre, Institute of Cardiovascular and Medial Sciences, University of Glasgow, 126 University Place, Glasgow, G12 8TA UK; 2 Department of Cardiology, Rigshospitalet University Hospital, Copenhagen, Denmark; 3 Division of Cardiovascular Medicine, Brigham and Women’s Hospital, Boston, MA USA; 4 Duke Clinical Research Institute, Duke University Medical Center, Durham, NC USA; 5 Population Health Research Institute, McMaster University, Hamilton, ON Canada; 6 Department of Molecular and Clinical Medicine, Sahlgrenska Academy, University of Gothenburg, Göteborg, Sweden; 7 National Heart and Lung Institute, Imperial College, London, UK

**Keywords:** Heart failure, Heart failure and preserved ejection fraction, Diabetes, Dysglycemia, Prognosis

## Abstract

**Purpose:**

The prevalence and consequences of prediabetic dysglycemia and undiagnosed diabetes is unknown in patients with heart failure (HF) and preserved ejection fraction (HFpEF) and has not been compared to heart failure and reduced ejection fraction (HFrEF).

**Methods:**

We examined the prevalence and outcomes associated with normoglycemia, prediabetic dysglycemia and diabetes (diagnosed and undiagnosed) among individuals with a baseline glycated hemoglobin (hemoglobin A1c, HbA1c) measurement stratified by HFrEF or HFpEF in the Candesartan in Heart failure Assessment of Reduction in Mortality and morbidity programme (CHARM). We studied the primary outcome of HF hospitalization or cardiovascular (CV) death, and all-cause death, and estimated hazard ratios (HR) by use of multivariable Cox regression models.

**Results:**

HbA1c was measured at baseline in CHARM patients enrolled in the USA and Canada and was available in 1072/3023 (35%) of patients with HFpEF and 1578/4576 (34%) patients with HFrEF. 18 and 16% had normoglycemia (HbA1c < 6.0), 20 and 22% had prediabetes (HbA1c 6.0–6.4), respectively. Finally among patients with HFpEF 22% had undiagnosed diabetes (HbA1c > 6.4), and 40% had known diabetes (any HbA1c), with corresponding prevalence among HFrEF patients being 26 and 35%. The rates of both clinical outcomes of interest were higher in patients with undiagnosed diabetes and prediabetes, compared to normoglycemic patients, irrespective of HF subtype, and in general higher among HFrEF patients. For the primary composite outcome among HFpEF patients, the HRs were 1.02 (95% CI 0.63–1.65) for prediabetes, HR 1.18 (0.75–1.86) for undiagnosed diabetes and 2.75 (1.83–4.11) for known diabetes, respectively, *p* value for trend across groups < 0.001. Dysglycemia was also associated with worse outcomes in HFrEF.

**Conclusions:**

These findings confirm the remarkably high prevalence of dysglycemia in heart failure irrespective of ejection fraction phenotype, and demonstrate that dysglycemia is associated with a higher risk of adverse clinical outcomes, even before the diagnosis of diabetes and institution of glucose lowering therapy in patients with HFpEF as well as HFrEF.

## Introduction

People with diabetes have a higher risk of developing incident heart failure (HF) than those without diabetes [1–3] and among patients with HF, those with diabetes have worse outcomes than patients without diabetes [4–6]. The latter observation is of considerable import because the prevalence of co-existing diabetes is high among patients with HF, affecting between 25 and 50% of individuals depending on which study is examined.

Recently, both undiagnosed diabetes and prediabetic dysglycemia were also found to be common in patients with HF with reduced ejection fraction (HFrEF) and each was associated with worse outcomes, compared with normoglycemia, although the risk was not as high as in patients with diagnosed diabetes [7]. The aim of our study was to determine the prevalence and prognostic significance of undiagnosed diabetes and prediabetic dysglycemia in the other major HF phenotype, HF with preserved ejection fraction (HFpEF), and compare the prevalence and outcomes with those in contemporaneously recruited patients with HFrEF. To do this, we used data from the Candesartan in Heart failure: Assessment of Reduction in Mortality and morbidity (CHARM) programme, which included patients with HFrEF and HFpEF [6, 8].

## Methods

The CHARM-Programme consisted of one HFpEF trial and two HFrEF trials. CHARM-Preserved enrolled 3023 patients 18 years or older, in York Heart Association (NYHA) functional class II–IV, with a prior hospitalization for a cardiac reason and a left ventricular ejection fraction (LVEF) above 40% [8]. CHARM-Alternative and CHARM-Added included 2028 and 2548 patients, respectively, aged 18 years or older, in NYHA functional class II-IV, with a LVEF of 40% or less, either treated with an angiotensin converting enzyme (ACE) inhibitor (CHARM-Added) or not, because of intolerance (CHARM-Alternative) [8]. As previously reported, glycated hemoglobin (hemoglobin A1c, HbA1c) was measured at baseline in the 2650 CHARM patients enrolled in Canada and the USA [6]. We categorized glycemic status in individuals without a history of diabetes using the International Diabetes Expert Committee criteria: normoglycemia (HbA1c < 6.0%), prediabetic dysglycemia (HbA1c 6.0–6.4%) and undiagnosed diabetes (HbA1c > 6.4%) [9]. Patients with a prior diagnosis of diabetes were considered to have diabetes irrespective of HbA1c level.

We used multivariable Cox proportional hazards models to evaluate the primary composite outcome of CHARM, which was death from cardiovascular (CV) causes or a hospitalization for HF, as well as all-cause mortality, according to glycemic status. Analyses were adjusted for age, sex, treatment arm, ejection fraction, NYHA class, heart rate, systolic blood pressure, body mass index (BMI), history of coronary artery bypass grafting, percutaneous coronary intervention, implantable cardioverter defibrillator, stroke and atrial fibrillation.

## Results

HbA1c was available in 1072/3023 (35%) of the patients in CHARM-Preserved and 428 patients with a HbA1c measurement in this trial had a history of diabetes (Table 1). Patients with diabetes were older, had a higher BMI, more evidence of coronary heart disease, worse NYHA class, higher heart rate, lower estimated glomerular filtration rate (eGFR) and greater use of loop diuretics compared to those with normoglycemia. Patients with previously undiagnosed diabetes and those with prediabetes had a clinical picture in between individuals with known diabetes and those with normoglycemia (Table 1).

**Table 1:** Baseline characteristics of patients with in CHARM-Preserved with HbA1c available, according to glycemic status

	No prior diagnosis of diabetes			Prior diabetes	*p* value
	HbA1c < 6.0	HbA1c 6.0–6.4	HbA1c > 6.4	Any HbA1c	
Patients, no (%)	189 (18%)	217 (20%)	238 (22%)	428 (40%)	
Age, years	63 ± 12	67 ± 11	69 ± 11	65 ± 11	< 0.001
Female, *n* (%)	82 (43%)	94 (43%)	100 (42%)	187 (44%)	0.98
HbA1c, median (Q1–Q3)	5.6 (5.5–5.7)	6.1 (6.0–6.2)	6.7 (6.5–7.1)	7.8 (7.1–9.0)	< 0.001
NYHA class, *n* (%)					0.0006
II	105 (56%)	120 (55%)	115 (48%)	168 (39%)	
III	81 (43%)	94 (43%)	119 (50%)	246 (57%)	
IV	3 (2%)	3 (1%)	4 (2%)	14 (3%)	
Ejection fraction	0.56 ± 0.10	0.55 ± 0.09	0.55 ± 0.09	0.54 ± 0.09	0.25
Heart rate, bpm	68 ± 11	69 ± 11	70 ± 12	72 ± 11	< 0.001
SBP, mmHg	132 ± 19	133 ± 17	133 ± 18	134 ± 17	0.86
BMI, kg/m^2^	29.4 ± 6.4	29.8 ± 6.3	29.8 ± 6.8	32.9 ± 7.1	< 0.001
eGFR, ml/min/1.73m^2^	82 ± 22	74 ± 23	70 ± 25	69 ± 27	< 0.001
Medical history, *n* (%)					
Ischemic etiology	81 (43%)	108 (50%)	122 (51%)	241 (56%)	< 0.001
Prior CABG	39 (21%)	59 (27%)	67 (28%)	144 (34%)	< 0.001
Prior PCI	33 (17%)	43 (20%)	52 (22%)	94 (22%)	0.59
Prior stroke	16 (8%)	24 (11%)	19 (8%)	53 (12%)	0.25
Prior AF	51 (27%)	73 (34%)	87 (37%)	121(28%)	0.07
Loop diuretic	110 (58%)	144 (66%)	162 (68%)	319 (75%)	< 0.001
Digoxin	56 (30%)	68 (31%)	87 (37%)	158 (37%)	0.21
β-blocker	106 (56%)	123 (57%)	129 (54%)	243 (57%)	0.93
MRA	17 (9%)	22 (10%)	18 (8%)	52 (12%)	0.27

*HbA1c* hemoglobin A1c, *NYHA* New York Heart Association functional class, *SBP* systolic blood pressure, *BMI* body mass index, *eGFR* estimated glomerular filtration rate, *CABG* coronary artery bypass graft, *PCI* percutaneous coronary intervention, *AF* atrial fibrillation, *MRA* mineralocorticoid receptor antagonist

HbA1c was available in 1578/4576 (34%) of individuals in CHARM-Alternative and CHARM-Added, and 558 patients in these trials with a HbA1c measurement had a history of diabetes (Table 2). Similar to what was observed in HFpEF, we found that patients with known or undiagnosed diabetes were older, had a worse NYHA class distribution and kidney function and were more likely to have evidence of coronary heart disease.

**Table 2:** Baseline characteristics of patients with in CHARM-Added/Alternative with HbA1c available, according to glycemic status

	No prior diagnosis of diabetes			Prior diabetes	*p* value
	HbA1c < 6.0	HbA1c 6.0–6.4	HbA1c > 6.4	Any HbA1c	
Patients, no (%)	254 (16%)	349 (22%)	417 (26%)	558 (35%)	
Age, years	61 ± 13	65 ± 12	67 ± 11	64 ± 10	< 0.001
Female, *n* (%)	67 (26%)	93 (27%)	108 (26%)	152 (27%)	0.97
HbA1c, median (Q1–Q3)	5.6 (5.4–5.7)	6.1 (6.0–6.2)	6.7 (6.5–7.1)	8.1 (7.1–9.3)	< 0.001
NYHA class, *n* (%)					0.028
II	93 (37%)	104 (30%)	124 (30%)	139 (25%)	
III	155 (61%)	238 (68%)	278 (67%)	398 (71%)	
IV	6 (2%)	7 (2%)	15 (4%)	21 (4%)	
Ejection fraction	0.28 ± 0.08	0.29 ± 0.09	0.27 ± 0.08	0.27 ± 0.08	< 0.001
Heart rate, bpm	71 ± 12	72 ± 12	72 ± 12	75 ± 12	< 0.001
SBP, mmHg	125 ± 18	124 ± 19	123 ± 19	126 ± 19	0.09
BMI, kg/m^2^	27.9 ± 4.9	27.5 ± 5.7	28.1 ± 5.7	30.3 ± 6.1	< 0.001
eGFR, ml/min/1.73m^2^	81 ± 23	72 ± 24	68 ± 25	67 ± 26	< 0.001
Medical history, *n* (%)					
Ischemic etiology	134 (53%)	212 (61%)	268 (64%)	390 (70%)	< 0.001
Prior CABG	78 (31%)	112 (32%)	142 (34%)	216 (39%)	0.08
Prior PCI	42 (17%)	57 (16%)	80 (19%)	137 (25%)	0.007
Prior stroke	19 (7%)	31 (9%)	54 (13%)	63 (11%)	0.091
Prior AF	62 (24%)	92 (26%)	128 (31%)	149 (27%)	0.29
Loop diuretic	174 (69%)	258 (74%)	333 (80%)	460 (82%)	< 0.001
Digoxin	165 (65%)	222 (64%)	267 (64%)	394 (71%)	0.07
β-blocker	136 (54%)	163 (47%)	232 (56%)	330 (59%)	0.003
MRA	44 (17%)	52 (15%)	93 (22%)	100 (18%)	0.06

*HbA1c* hemoglobin A1c, *NYHA* New York Heart Association functional class, *SBP* systolic blood pressure, *BMI* body mass index, *eGFR* estimated glomerular filtration rate, *CABG* coronary artery bypass graft, *PCI* percutaneous coronary intervention, *AF* atrial fibrillation, *MRA* mineralocorticoid receptor antagonist

Only 18% of patients with HFpEF and 16% of patients with HFrEF were normoglycemic.

Prediabetes was more common than normoglycemia in both types of HF: 20% in patients with HFpEF and 22% in those with HFrEF (*p* = 0.25).

The prevalence of undiagnosed diabetes was also high, but was less common in patients with HFpEF compared with HFrEF (22 vs. 26%, *p* = 0.01). Conversely, the prevalence of known diabetes was higher in patients with HFpEF (40 vs 35%, *p* = 0.02). As a result, the prevalence of any diabetes (diagnosed and previously undiagnosed) was 62% in each study.

**Fig. 1:**
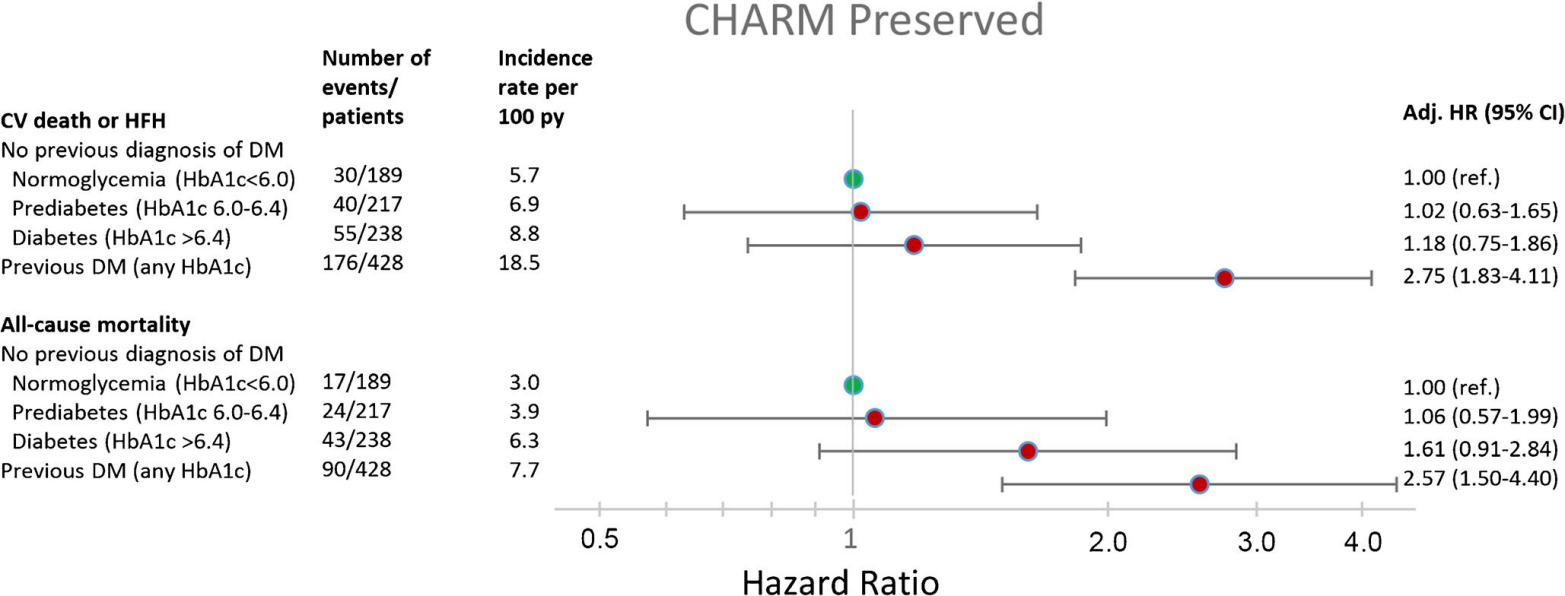
Adjusted risk for the primary composite outcome and all-cause mortality in CHARM-Preserved for each glycemia category

**Fig. 2:**
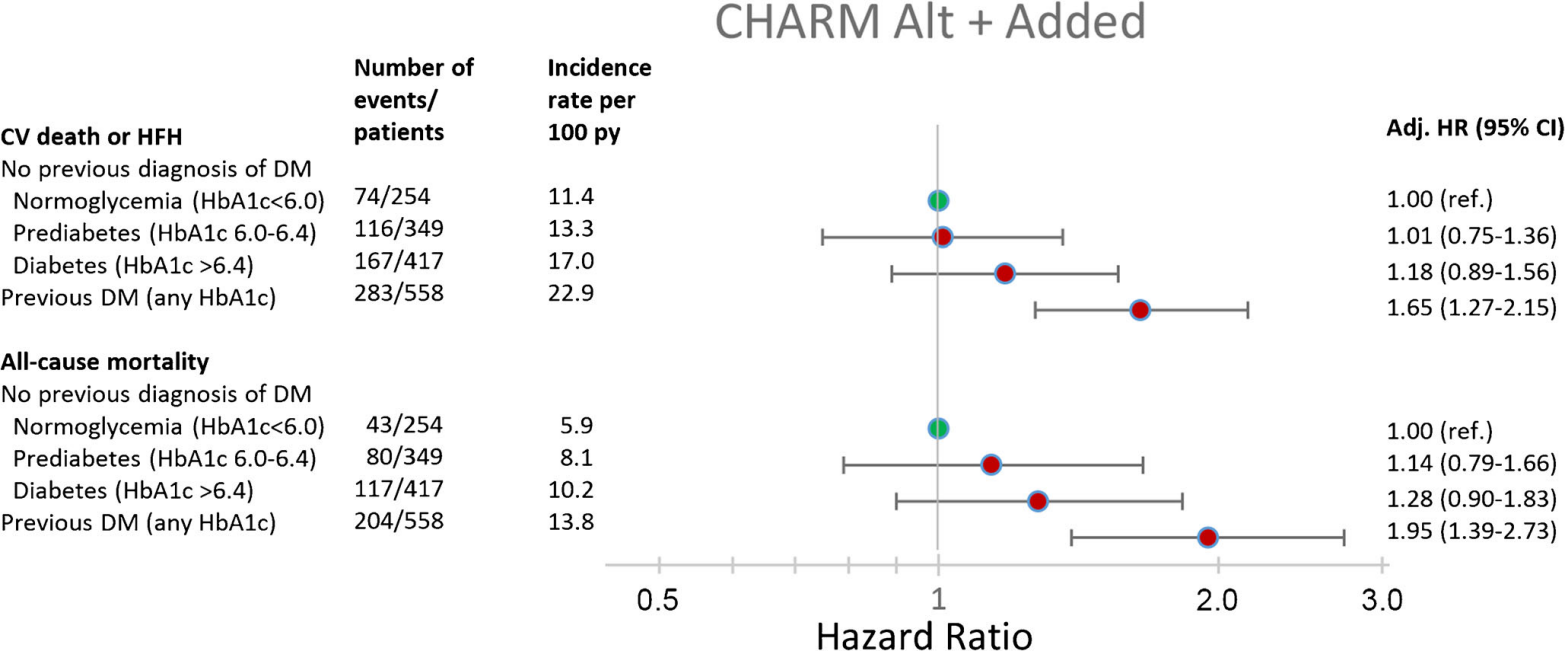
Adjusted risk for the primary composite outcome and all-cause mortality in CHARM-Alternative/Added for each glycemia category

HFpEF and HFrEF patients with diagnosed diabetes were at significantly higher risk of both the primary composite outcome, and all-cause mortality, compared with normoglycemic patients (Figs. 1 and 2). The rates of both outcomes of interest were higher in patients with undiagnosed diabetes and prediabetes, compared with normoglycemic patients, *p* < 0.001 for trend across dysglycemia categories for both HFpEF and HFrEF.

## Discussion

We found that the prevalence of diagnosed diabetes was higher in patients with HFpEF (40%) in CHARM than in those with HFrEF (35%). However, the novel data in the present report relate to the prevalence of undiagnosed diabetes and prediabetic dysglycemia, each of which were more common than a normal HbA1c in both HFpEF and HFrEF. Indeed, when undiagnosed and diagnosed diabetes were combined, a remarkable 62% of patients with each type of HF had diabetes. An additional fifth or so of patients had prediabetes, leaving just approximately one in six patients with a normal HbA1c, an observation that was true for both HFrEF and HFpEF. In comparison 26% had a normal HbA1c and 49% had either diagnosed or undiagnosed diabetes in the Prospective Comparison of ARNI with ACEI to Determine Impact on Global Mortality and Morbidity in Heart Failure (PARADIGM-HF), and corresponding numbers were 30 and 41%, respectively, in the Gruppo Italiano per lo Studio della Sopravvivenza nella Insufficienza Cardiaca-Heart Failure (GISSI-HF) trial [10]. One explanation for the higher prevalence in CHARM (irrespective of HF type) is that HbA1c was only measured in North American patients where the prevalence of diabetes is high, with the latest reports estimating that in the general population almost 10% have diagnosed or undiagnosed diabetes and another 34% of the population have prediabetes [11].

As was recently reported for patients with HFrEF, undiagnosed diabetes and prediabetic dysglycemia in patients with HFpEF (and HFrEF) in the present study were associated with worse outcomes than observed in patients with a normal HbA1c. Although this pattern was very similar to that reported in the PARADIGM-HF trial, no elevated risk was reported for patients with prediabetes in GISSI-HF. The difference in risk in the present study was not statistically significant, probably because of the smaller number of subjects with a HbA1c measurement in CHARM [7]. Although we found relative similar prevalences of dysglycemia in patients with HFpEF and HFrEF in the current study, the patients with these two phenotypes differed in respect of many of their baseline characteristics. Patients with HFrEF were more likely to have ischemic etiology, worse NYHA class and were less likely to be female. To some extent, the finding of a similarly high rate of dysglycemia in these two quite distinct phenotypes suggests that the syndrome of HF per se plays some role in the development of prediabetes and diabetes. Notably, insulin resistance is present in patients with idiopathic dilated cardiomyopathy as well as in those with ischemic cardiomyopathy, and it is greater in patients with coronary artery disease and HF than in patients with coronary artery disease without HF and is not correlated with ejection fraction [12–14]. These findings suggest that the high prevalence of dysglycemia in HF is not explained by recognized associations, e.g. with atherosclerosis and is related to HF per se, independently of ventricular function.

Like PARADIGM-HF, our study has the limitation of a single HbA1c value without a confirmatory measurement and the additional limitation of only including patients from North America where the prevalence of diabetes is higher than in other geographic regions, which may introduce a selection bias and impair applicability of our results to other regions of the world.

However, even with these limitations, our data confirm the remarkably high prevalence of dysglycemia in HFrEF and show that a similarly high prevalence is found in HFpEF (with only 16–18% of patients having a normal HbA1c). In both types of HF, dysglycemia is associated with a higher risk of adverse clinical outcomes, even before the diagnosis of diabetes. These findings raise questions about the potential value of screening (for undiagnosed diabetes) and treatment targeted at correcting dysglycemia in patients with both HF phenotypes [15].
